# Worldwide Prevalence of Hearing Loss Among Smartphone Users: Cross-Sectional Study Using a Mobile-Based App

**DOI:** 10.2196/17238

**Published:** 2020-07-23

**Authors:** Marcin Masalski, Krzysztof Morawski

**Affiliations:** 1 Department of Otolaryngology Head and Neck Surgery Faculty of Medicine Wroclaw Medical University Wroclaw Poland; 2 Department of Biomedical Engineering Faculty of Fundamental Problems of Technology Wroclaw University of Technology Wroclaw Poland

**Keywords:** hearing loss, epidemiology, mobile-based, hearing test, pure-tone audiometry

## Abstract

**Background:**

In addition to the aging process, risk factors for hearing loss in adults include, among others, exposure to noise, use of ototoxic drugs, genetics, and limited access to medical care. Differences in exposure to these factors are bound to be reflected in the prevalence of hearing loss. Assessment of hearing loss can easily be carried out on a large scale and at low cost using mobile apps.

**Objective:**

This study aimed to conduct a worldwide assessment of the differences in hearing loss prevalence between countries in a group of mobile device users.

**Methods:**

Hearing tests were conducted using the open-access Android-based mobile app Hearing Test. The app is available free of charge in the Google Play store, provided that consent to the use of the results for scientific purposes is given. This study included hearing tests carried out on device models supported by the app with bundled headphones in the set. Calibration factors for supported models were determined using the biological method. The tests consisted of self-determining the quietest audible tone in the frequency range from 250 Hz to 8 kHz by adjusting its intensity using the buttons. The ambient noise level was optionally monitored using a built-in microphone. Following the test, the user could compare his hearing threshold against age norms by providing his or her age. The user's location was identified based on the phone’s IP address.

**Results:**

From November 23, 2016 to November 22, 2019, 733,716 hearing tests were conducted on 236,716 mobile devices across 212 countries. After rejecting the tests that were incomplete, performed with disconnected headphones, not meeting the time criterion, repeated by the same user, or carried out regularly on one device, 116,733 of 733,716 tests (15.9%) were qualified for further analysis. The prevalence of hearing loss, defined as the average threshold at frequencies 0.5 kHz, 1 kHz, 2 kHz, and 4 kHz above 25 dB HL in the better ear, was calculated at 15.6% (95% CI 15.4-15.8). Statistically significant differences were found between countries (*P*<.001), with the highest prevalences for Bangladesh, Pakistan, and India (>28%) and the lowest prevalences for Taiwan, Finland, and South Korea (<11%).

**Conclusions:**

Hearing thresholds measured by means of mobile devices were congruent with the literature data on worldwide hearing loss prevalence. Uniform recruitment criteria simplify the comparison of the hearing loss prevalence across countries. Hearing testing on mobile devices may be a valid tool in epidemiological studies carried out on a large scale.

## Introduction

The worldwide prevalence of hearing loss is estimated to be between 4.0% and 18.1% [1-5], depending on the methodology, in particular the degree of hearing impairment used in the definition of hearing loss. Methodological differences also include the selection of data sources and calculation methods. Despite the uncertainty of the estimation, the scale of the burden is significant. Hearing loss is also an important problem because of its side effects. Due to communication disorders, hearing loss leads to decreased social activity, lower self-esteem, and consequently, stigmatization, social exclusion, and depression [6,7].

Apart from age, risk factors for hearing loss in adults include, but are not limited to, exposure to noise, use of ototoxic drugs, genetic conditions, infectious diseases, and limited access to medical care [5,8-12]. In view of increasing exposure to risk factors for hearing loss, in particular the aging population and increased exposure to noise in developing countries, the number of people with hearing loss has increased over the years [4,5,12].

Epidemiological analysis of hearing loss allows the identification of causes and planning of remedies [4,12]. Activities in the field of prevention and treatment of hearing loss are highly recommended, as it is estimated that 50% of hearing loss cases can be prevented and a significant portion of the remaining cases can be treated [4].

Worldwide geo-epidemiological investigations of hearing loss have been conducted in the form of meta-analyses of available data from different studies as a consequence of political, financial, cultural, and geographical limitations in the planning of uniform research on such a large scale [1-3,13]. Owing to the differences in study settings, in particular the differences in the definition of hearing loss, age groups, and method of participant recruitment, direct comparison of results is not possible, whereas indirect comparison is burdened with uncertainty resulting from sparse data adjustment [1-3,13].

Self-performed hearing tests on mobile devices can be easily carried out on a large scale and at low cost while maintaining a uniform qualification criterion. They are broadly accessible due to widespread use of smartphones, and they do not require engaging qualified personnel, while their results are comparable to pure-tone audiometry [14-23]. The difference in hearing threshold between the self-test in the Hearing Test app and pure-tone audiometry is estimated at 2.6 dB (SD 8.3 dB) [24], and the absolute difference is estimated at 8.8 dB [17].

The aim of this study was to assess the worldwide prevalence of hearing loss among Android users by means of the Hearing Test app [24,25] and compare the prevalence across countries.

## Methods

### Ethical Concerns

This was a cross-sectional study of hearing loss prevalence carried out by means of the open-access, mobile-based Hearing Test app [26]. Subject consent to use the results for scientific purposes was required the first time the app was started. The consent to research was issued by the Bioethics Committee at the Medical University in Wroclaw. Evaluation of the app, rationale behind the app, and comparison with other studies concerning mobile and web-based pure-tone hearing screening have been presented in prior articles [24,25,27,28].

### Recruitment

The participants were recruited via the Hearing Test app that runs on Android systems. The app is offered free of charge in the Google Play store, where the user can become familiar with the app's features and download it to a phone or tablet. Thus, eligibility criteria for participants only included access to an Android device that is supported by the app. The test could be carried out by the phone owner or with his or her assistance.

### Device Calibration

The study included tests carried out on mobile devices that supported the Hearing Test app for which calibration coefficients had been determined. All the tests in the study were carried out on bundled headphones that were supplied by the manufacturer in a set with the device. Devices of the same model used common calibration coefficients that were determined by the biological method [27,28] on the basis of at least 16 measurements carried out by subjects with normal hearing on different devices of the model [25]. This method is characterized by the standard error of determining the calibration coefficients below 5 dB and within-model variability at 4 dB [25]. The headphone connection status was monitored by the app, whereas the usage of bundled headphones was confirmed by the user. Tests taken using headphones other than bundled headphones were not taken into account.

### Hearing Threshold

The hearing test consisted of self-determining the quietest audible sound by adjusting its intensity using the “I can hear” and “I can't hear” buttons. The intensity of a test tone could be changed many times. The quietest audible sound was confirmed with the “Barely audible” button. A modulated test tone was used, with a 100% modulation depth, 2 Hz modulation frequency, and an intensity changed in steps of 5 dB. When the intensity of the tone exceeded a 40 dB hearing level (HL), a narrowband masking noise was generated contralaterally at an intensity of 40 dB HL. For test tones above 60 dB HL, the intensity of the masking noise was increased to 60 dB HL. By default, the tests were carried out for frequencies from 250 Hz to 8 kHz; however, this range could be changed in the settings. The time to determine the hearing threshold was measured separately for each frequency. The hearing threshold determined in this way has been compared in previous works with pure-tone audiometry. A difference was found at the level of 2.6 dB (SD 8.3) [24], and the absolute difference was found at 8.8 dB [17].

### Ambient Noise

Ambient noise was monitored during the measurement after obtaining the user's consent to access the microphone resources. An equivalent continuous A-weighted sound pressure level LAeq was registered in accordance with the Android specification [29] that sets a reference point at 90 dB SPL.

### Age

At the end of the test, the users could optionally enter their age to compare the obtained hearing threshold against the age-based norm. By design, the app was developed for adults. Therefore, the user chose his or her age by selecting a value from 18-90 years. Providing the age was not obligatory for this study, to minimize the amount of potentially incorrect data. All the entered age values were recorded in the database to eliminate tests for which several different values were given. The age was also determined from the note, where it could optionally be given. User gender was not collected.

### Geolocalization

The user's country was determined using the geoPlugin service [30] on the basis of the IP address.

### Statistical Analysis

#### Power

Comparison of hearing loss prevalence between countries was conducted by adopting the definition of hearing loss as the average hearing threshold at frequencies 0.5 kHz, 1 kHz, 2 kHz, and 4 kHz above 25 dB HL in the better ear. The minimum number of tests for the country to be included in the analysis was calculated based on the standard deviation of the average hearing threshold determined in preliminary measurements at 17 dB. Assuming a level of statistical significance of .05, test power of .8, and an effect size of 5.0 dB, a sample size of 90 tests was obtained.

#### Data Exclusion

The tests in this study were unsupervised. Therefore, prior to analysis, they were verified for duration. A very short duration suggests unreliable results that can be manifested by an increased measurement error. To eliminate these tests, the standard deviation of the hearing threshold grand average was analyzed in relation to the test duration. Time threshold was set at the level at which stabilization of the standard deviation was observed. Incomplete tests, test carried out without headphones connected, and tests repeated on the same device were excluded as well.

#### Data Analysis

Both the subject’s age and level of ambient noise during the test belong to data for which acquisition required additional action on the part of the user. Preliminary analyses have shown that these are sparse data compared to the hearing threshold, geolocation, and device model. Therefore, the country-specific hearing loss prevalence was calculated based on all the tests, whereas the bias was estimated and discussed basing on subgroup analysis. The tests with age were used to characterize the population and analyze an age-related hearing threshold, while tests with noise were used to calculate the ambient noise effect. Distributions of hearing thresholds in the subgroups were tested for equivalence using the Kolmogorov-Smirnov test. Confidence intervals for the hearing threshold median and the hearing loss prevalence were determined by means of bootstrapping.

## Results

### Data Collection and Exclusion

**Figure 1:**
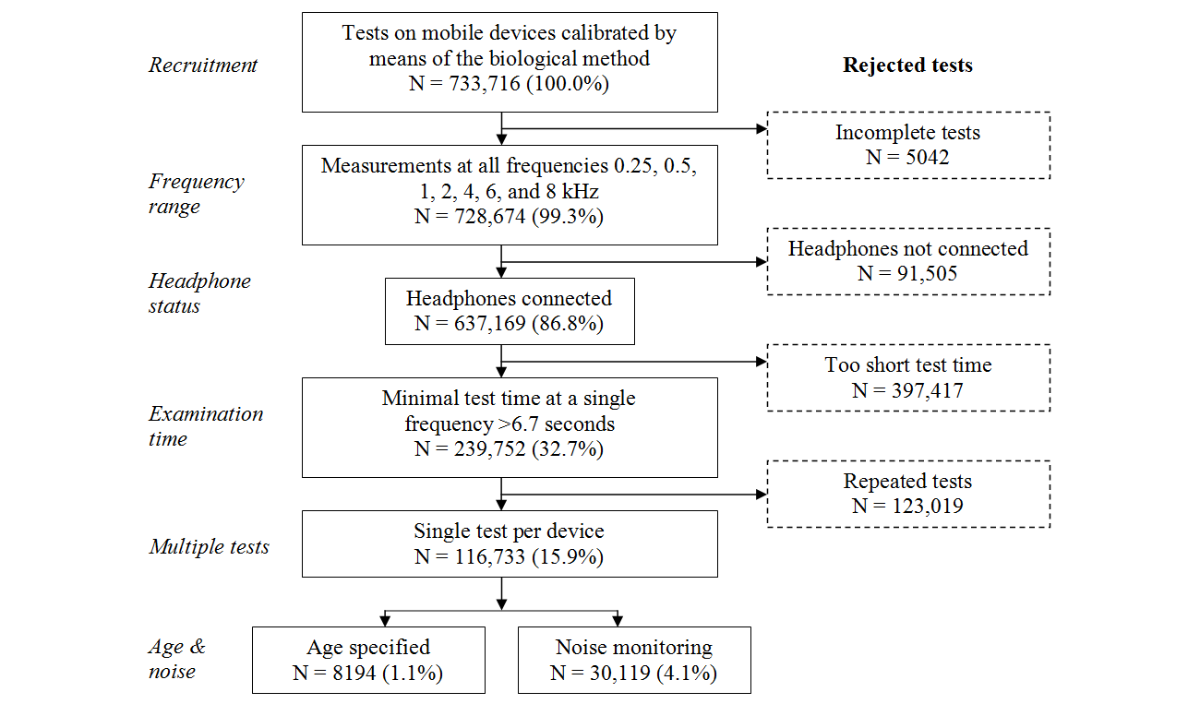
Flow diagram.

In the period from November 23, 2016 to November 22, 2019, 733,716 tests were carried out on 236,716 devices in 212 countries. The hearing threshold measurement at the fundamental frequencies 0.25 kHz, 0.5 kHz, 1 kHz, 2 kHz, 4 kHz, 6 kHz, and 8 kHz was performed in 728,674 of 733,716 (99.3%) tests. After examining headphone connection status and rejecting the tests carried out with disconnected headphones, 637,169 of 733,716 (86.8%) tests were obtained (Figure 1).

**Figure 2:**
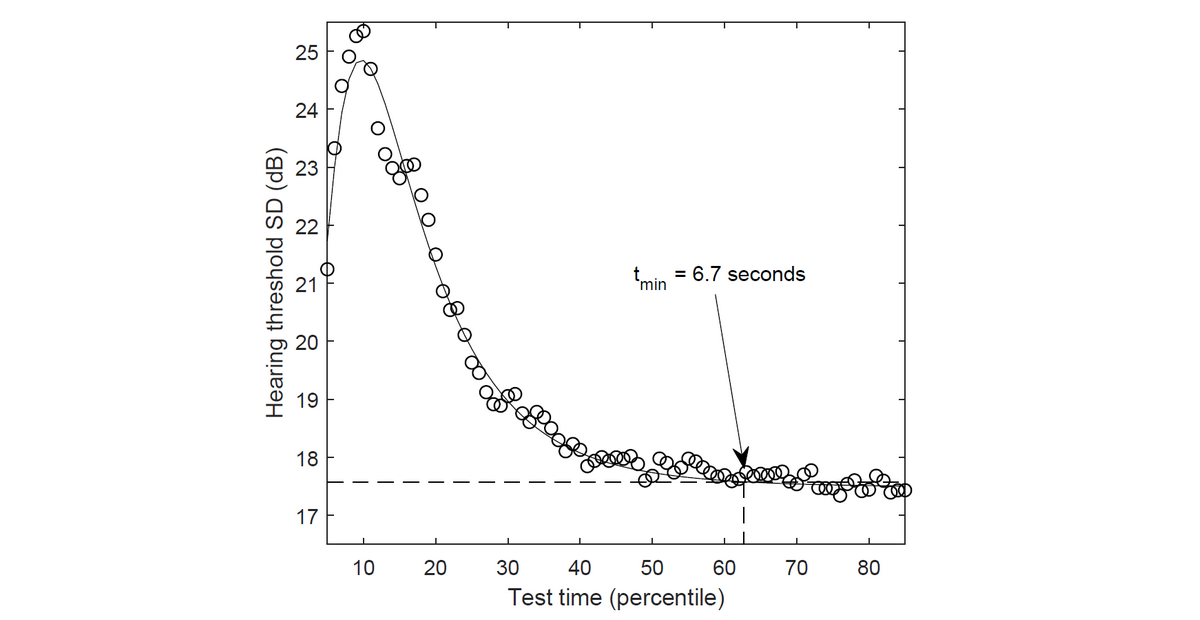
Grand standard deviation of the hearing threshold in relation to the duration of the measurement at a single frequency.

Time limits were introduced to discard tests with a duration that actually prevented a correct measurement. The grand standard deviation of the hearing threshold in relation to the duration of the measurement at a single frequency is presented in Figure 2. Stabilization of the deviation is observed at longer durations. It has been assumed that tests in which the measurement at any frequency was carried out quicker than 6.7 seconds are subject to significant error. This threshold was set heuristically by fitting the data with a lognormal distribution and assuming its value at a cumulative probability density level of 0.99 (Figure 2). The time requirements were met by 239,752 of 733,716 (32.6%) tests.

Many devices were used for more than one test. To eliminate tests repeated by the same person or carried out in bulk (eg, in outpatient clinics or as part of screening programs) only one test from each device was qualified for the analysis. The last test was selected, at first considering the test with an age provided. Finally, 116,733 of 733,716 (15.9%) tests were obtained for the analysis. Age was provided for 8194 of 116,733 (7.0%) tests, and monitoring for ambient noise was present in 30,119 of 116,733 (25.8%) tests, whereas only 2556 of 116,733 (2.2%) tests included both age and noise data.

### Age

The subject's age was provided for 8194 of 116,733 (7.0%) tests that qualified for the analysis. The age characteristics of the research group is shown in Table 1. The age group with the highest number of tests was 30-39 years, while the fewest subjects were in the oldest groups (80-89 years and >89 years). The median age was 39 years, and the average age was 40.0 years.

Tests with age provided were compared with those without age provided to estimate the bias resulting from the generalization of the results for the whole group. There were no statistically significant differences between the distributions of the hearing thresholds at the level of *P*=.05.

**Table 1:** Ages of the participants (n=8194).

Age characteristics		Participant values
Age (years), mean (SD)		40.0 (15.1)
Age (years), median		39
**Age (years), n (%)**		
	<20	842 (10.3)
	20-29	1597 (19.5)
	30-39	1813 (22.1)
	40-49	1725 (21.1)
	50-59	1227 (15.0)
	60-69	655 (8.0)
	70-79	253 (3.1)
	80-89	62 (0.8)
	>89	20 (0.2)

### Hearing Threshold

**Figure 3:**
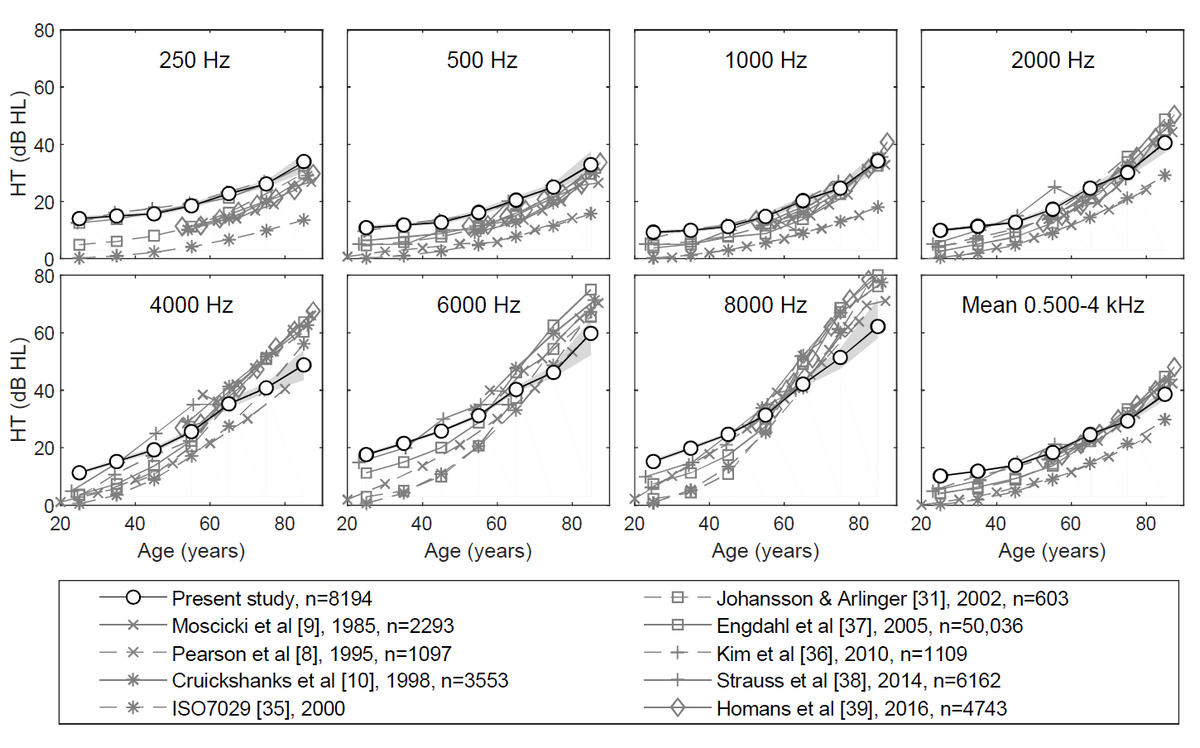
Hearing threshold by age group and frequency. Dashed lines indicate screening studies including screening only for noise exposure [31], and continuous lines show studies without screening. The grey area illustrates the 99% CI.

Hearing threshold in relation to age was analyzed based on all 7332 of 116,733 (6.3%) tests for which the age provided was within the range of 20 to 89 years. Tests completed by subjects aged 18, 19, or 90 years were omitted from the analysis to obtain equal age ranges (20-29 to 80-89 years). Moreover, the ages of 18 and 90 years were characterized by a higher number of tests in comparison with the adjacent age groups, suggesting the presence of additional data, such as results for subjects younger than 18 years or older than 90 years, or with incorrectly provided data. The median thresholds by age group and frequency are given in Figure 3 and in Multimedia Appendix 1. A comparison with the prior work presented in Figure 3 is provided in the Discussion section.

### Country Prevalence of Hearing Loss

**Figure 4:**
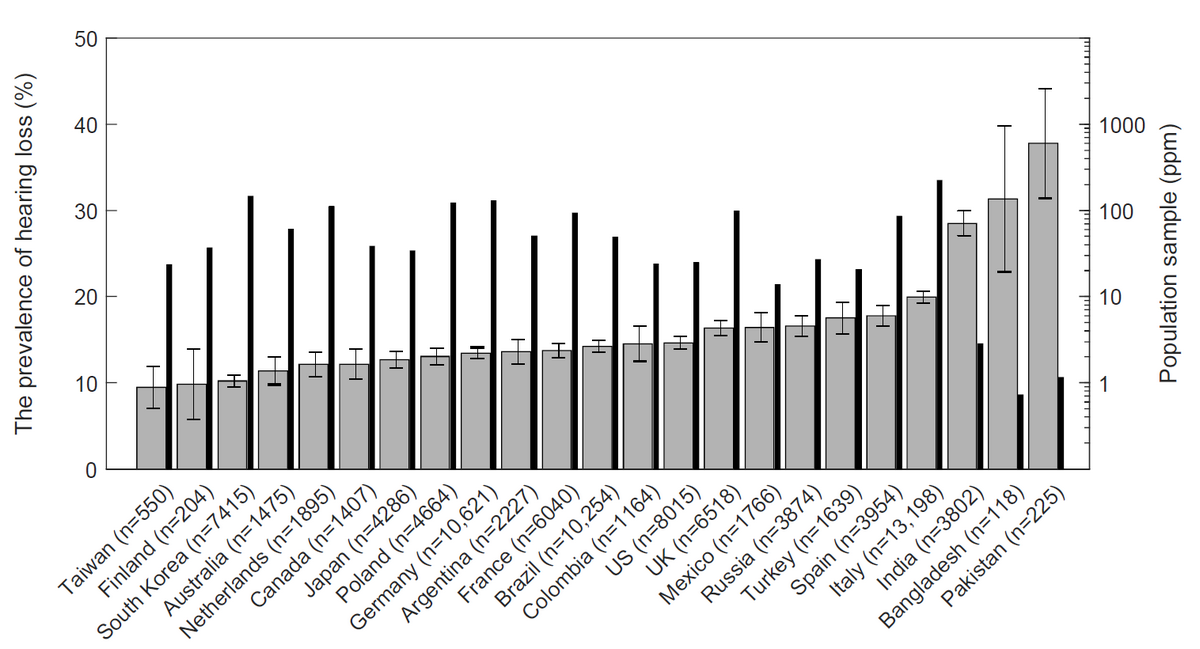
The prevalence of hearing loss by country. Hearing loss was defined as an average hearing threshold >25 dB hearing level (HL) in the better ear at frequencies of 0.5 kHz, 1 kHz, 2 kHz, and 4 kHz. Whiskers indicate 95% CI. Countries with >1000 tests or with boundary values of hearing loss prevalence are included. The full list is available in Multimedia Appendix 2. ppm: number of individuals per million people in the population.

**Figure 5:**
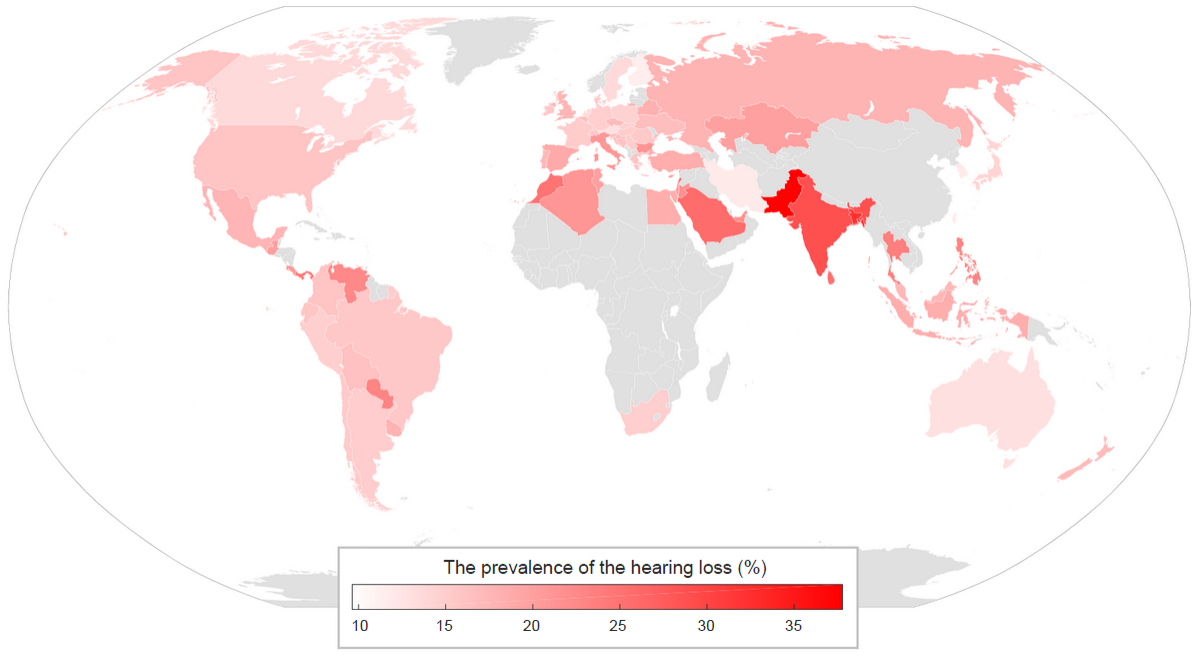
Global prevalence map of hearing loss.

The prevalence of hearing loss was determined based on 733,716 tests conducted in 212 countries. The required sample size of 90 tests was reached for 74 countries. The prevalence of hearing loss, defined as a mean hearing threshold >25 dB HL in the better ear at 0.5-4 kHz, is presented in Figure 4, Figure 5, and Multimedia Appendix 2. The highest prevalences were obtained for Pakistan, Bangladesh, and India at 37.8% (95% CI 31.4-44.2), 32.2% (95% CI 23.6-40.8), and 28.5% (95% CI 27.1-29.9), respectively, whereas the lowest prevalences were obtained for Taiwan, Finland, and South Korea at 9.6% (95% CI 7.2-12.1), 9.8% (95% CI 5.7-13.9), and 10.2% (95% CI 9.5-10.9), respectively. The global prevalence of hearing loss was calculated at 15.6% (95% CI 15.4-15.8).

### Effect of Ambient Noise

**Figure 6:**
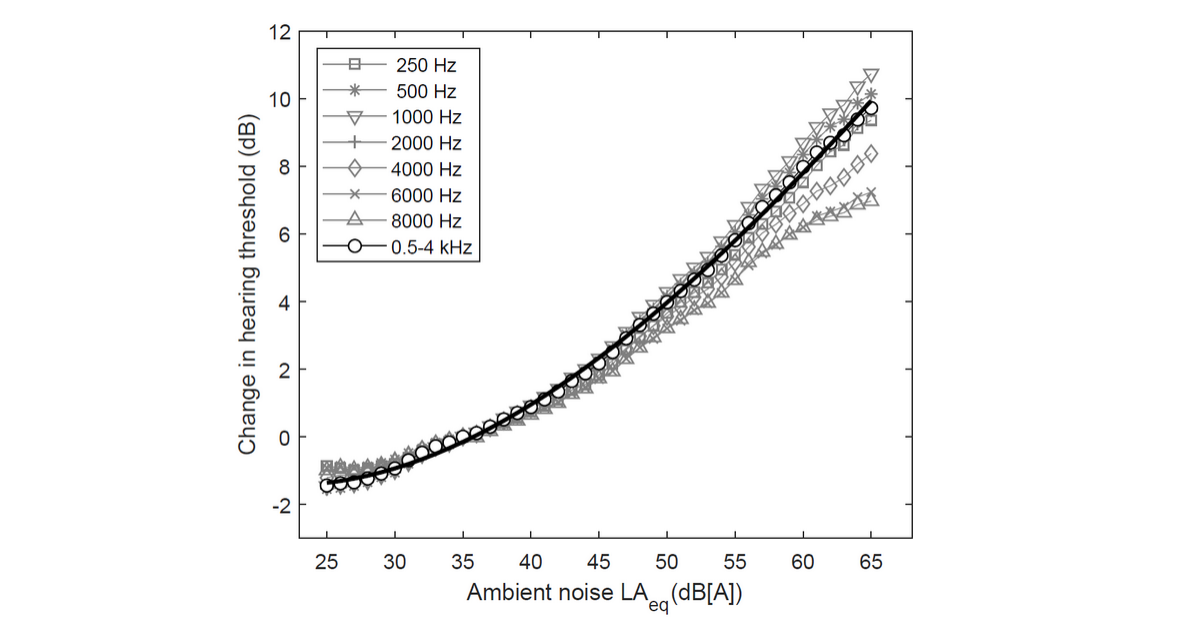
The difference in hearing threshold in relation to ambient noise level.

The effect of ambient noise was estimated based on 30,119 of 116,733 (25.8%) tests conducted with ambient noise monitoring. The difference in the hearing threshold in relation to the ambient noise level is presented in Figure 6. Hearing thresholds were adjusted for ambient noise by assuming the reference level LAeq=35 dB(A) [32]. The average decrease in the hearing threshold was obtained at a level of 2.53 dB (SD 0.74 dB). Additionally, the distributions of the hearing threshold in the groups with and without ambient noise monitoring turned out to be significantly different at the level of *P*<.001, with the monitoring group having a hearing threshold that was lower by 1.25 dB (SD 0.12 dB). This produces an overall effect of ambient noise of 3.78 dB (SD 0.75 dB), which corresponds to a 4.16% (SD 1.46%) reduction in the country-specific prevalence.

The highest ambient noise levels were found in Pakistan, Bangladesh, and India, which corresponded to 10.7%, 8.6%, and 6.2% decreases, respectively, in the prevalences of hearing loss. Nevertheless, these countries still had the highest prevalences of hearing loss.

### Effect of the Device Model

Differences in hearing thresholds between device models were found to be significant at the level of *P*=.01. These differences result directly from the biological calibration method that is based on determination of the reference sound level in relation to subjects with normal hearing. The standard error of the biological calibration estimated at 4.2 dB for 16 independent measurements [25] justifies the discrepancies. After adjusting for the device model, the mean hearing threshold by country changed by a mere −0.07 dB (SD 0.96 dB).

## Discussion

### Principal Findings

This paper presents the prevalence of hearing loss worldwide based on the 116,733 hearing tests conducted by Android users on mobile devices. The global prevalence of hearing loss was 15.6% (95% CI 15.4-15.8). Statistically significant differences were found across countries (*P*<.001). Of the 212 countries, 74 countries exceeded the number of tests required for the assumed sample size. Amongst these countries, the highest prevalences were found in Pakistan, Bangladesh, and India at 37.8% (95% CI 31.4-44.2), 32.2% (95% CI 23.6-40.8), and 28.5% (95% CI 27.1-29.9), respectively, whereas the lowest prevalences were found in Taiwan, Finland, and South Korea at 9.6% (95% CI 7.2-12.1), 9.8% (95% CI 5.7-13.9), and 10.2% (95% CI 9.5-10.9), respectively. The risk of hearing loss in Pakistan was 74.6% (95% CI 38.2-110.7) higher than in Taiwan. The absolute difference was 28.2% (95% CI 14.4-41.8).

Of the 733,716 tests, 116,733 (15.9%) were selected for the analysis. The number of tests rejected due to incompleteness, short duration, lack of headphones, or repetitions on a single device seems reasonable, especially as prior to performing a proper test, users often become familiar with the app by conducting a trial test.

In addition to Finland, relatively low hearing loss values were obtained for the other two Scandinavian countries, Sweden and Norway, at 12.1% (95% CI 8.4-15.9) and 12.7% (95% CI 8.1-17.4), respectively. The prevalences of hearing loss for the other two Asian Tigers (ie, Singapore and Hong Kong) were slightly higher, at 14.6% (95% CI 10.0-19.2) and 15.3% (95% CI 10.3-20.4), respectively. The high prevalence of hearing loss in India, which, along with Pakistan and Bangladesh, is one of the most populated countries in the world, is supported by the findings of Garg et al [33]. Similar hearing loss prevalences were found in most Western and Central European countries, which were slightly higher than Australia (11.4%, 95% CI 9.8-13.0) and Canada (12.2%, 95% CI 10.4-13.9) but lower than that in the United States (14.6%, 95% CI 13.9-15.4). Similar prevalences of hearing loss were obtained among the countries of the former Soviet Union (ie, Russia, Belarus, Ukraine, and Kazakhstan), at levels between 15.4% (95% 12.9-17.9) for Ukraine and 18.9% (95% CI 14.3-23.6) for Kazakhstan.

In Italy, 20.0% (95% CI 19.3-20.7) of the subjects had hearing loss, and this value was definitely higher than in the other Western and Central European countries. The credibility of this result is questionable due to the much higher use of apps in this country, being the highest in the world and reaching 222.3 people per million population (Multimedia Appendix 2). Attention should also be paid to the low Iranian result of 10.3% (95% CI 7.8-12.7), which significantly differs from other countries of North Africa and the Middle East. On the other hand, according to a study by the World Health Organization [5] in the region of North Africa and the Middle East, the prevalence of hearing loss is lower than in developed countries.

The required sample size was not reached in a significant number of African countries. In the poorest countries, the limiting factor is access to mobile devices and the internet. The same situation may also occur in countries with significant social stratification, contributing to the lower prevalences of hearing loss resulting from a lack of data from the poorer part of the population. The lack of data from China is related to the lack of active Google Play service in this country.

The prevalence of hearing loss in countries has been correlated with the infant mortality rate [34], which is often used as an indicator of the health status in a country. A Cronbach alpha coefficient of 0.76 (95% CI 0.52-0.90) has been obtained.

### Comparison With Prior Work

#### Age-Related Hearing Loss

Age related hearing loss assessed by means of the mobile app was compared with the results from other studies [8-10,31,35-39] (Figure 3). The hearing threshold in subjects screened negatively for noise exposure [8,31,35-37], otologic disorders [8,35-37], ototoxic drugs [8,36], and asymmetric hearing [8,36,37] tends to be lower than that obtained in this study and prior unscreened studies [9,10,37-39]. However, substantial differences have been found between studies; although the threshold in the study by Kim et al [36] was higher than those in the studies by Engdahl et al [37] and Homans et al [39], it should have actually been lower because Kim et al conducted screening, while the other 2 studies did not [37,39]. In this study, for younger age groups (20-49 years), especially at low frequencies (250 Hz, 500 Hz), hearing thresholds were higher than in most previous studies. The results obtained at 250 Hz are consistent with those found by Kim et al [36] and Engdahl et al [37], while at 500 Hz, the results were consistent only with those found by Kim et al [36]. At frequencies above 4 kHz for older age groups (>70 years), the thresholds were lower than the values reported in the literature. Apart from the two youngest age groups (20-39 years) and the oldest age group (80-89 years), agreement with most studies was achieved for the average hearing threshold at the frequency range 0.5-4 kHz.

Increased hearing thresholds in younger age groups may be associated with a tendency for hearing-impaired subjects to take a test more willingly than people with normal hearing as well as with increased ambient noise, which mainly affects soft sounds. Lower values for the oldest groups might be related to an incorrect reaction to the masking noise. The effect of ambient noise and the masking noise is discussed in the Limitations section.

#### Hearing Loss in the World

**Figure 7:**
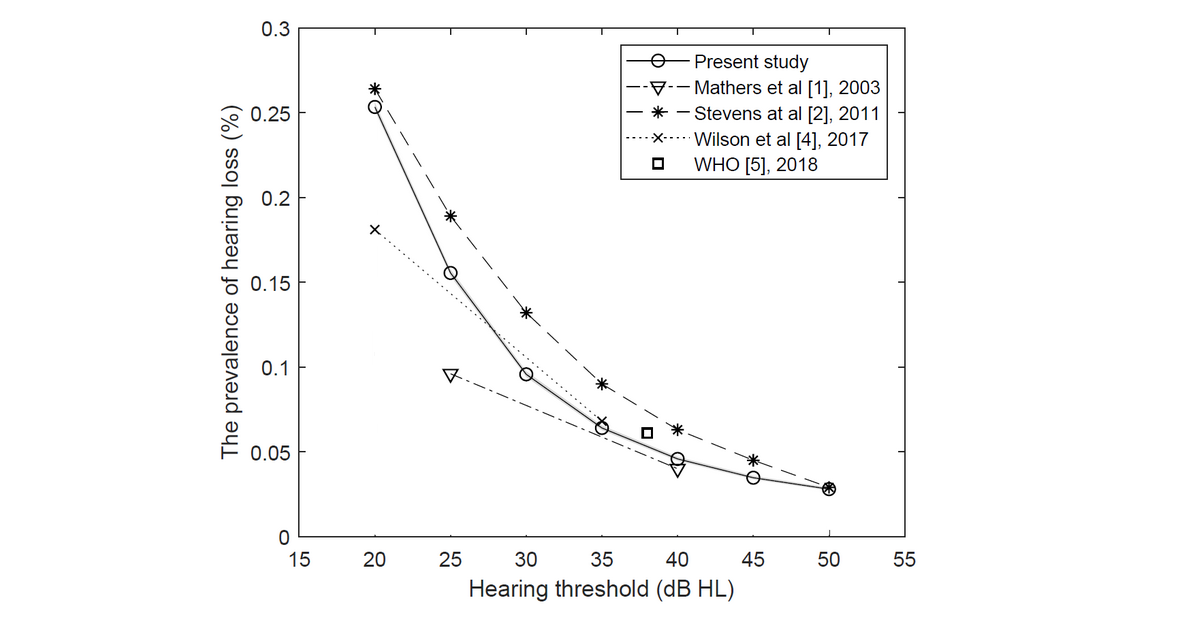
Worldwide prevalence of hearing loss with respect to the criterion based on the average hearing threshold in the better ear at frequencies 0.5-4 kHz. Separate thresholds for adults (40 dB hearing level [HL]) and children (30 dB HL) in the study by the World Health Organization [5].

The prevalence of hearing loss among users of the Hearing Test app may differ from population values, at least as regards the age structure. However, the value was compared with worldwide estimates. Figure 7 presents the worldwide prevalence of hearing loss with respect to the criterion based on the average hearing threshold in the better ear at frequencies 0.5-4 kHz. Despite statistically significant differences between the obtained results and other studies [1,2,4,5], the data are consistent.

#### Country-Specific Studies

The prevalence of hearing loss was also compared with other studies. Calculations were performed by adopting the criteria for hearing loss and matching age ranges (Multimedia Appendix 3). The prevalence of hearing loss was lower in previous studies [11,16,40-45], pursuant to at least one criterion in research [9,10,46,47], and was higher in other articles [39,48-52]. There is large variation within the data presented in the literature, which may result not only from age structure differences and heterogeneous hearing loss definitions but also from research methodology, especially trial-specific recruitment methods or exclusion criteria. Therefore, drawing conclusions regarding country differences in the prevalence of hearing loss by means of literature data is challenging, as it requires the use of estimation methods in the presence of sparse data [2,3,13]. In this aspect, for country comparison, mobile data seem more valuable than a meta-analysis, although other confounding factors such as cultural background, propensity to new technologies, economic status, or app promotion should be underlined here as well.

### Limitations

#### Masking Noise

**Figure 8:**
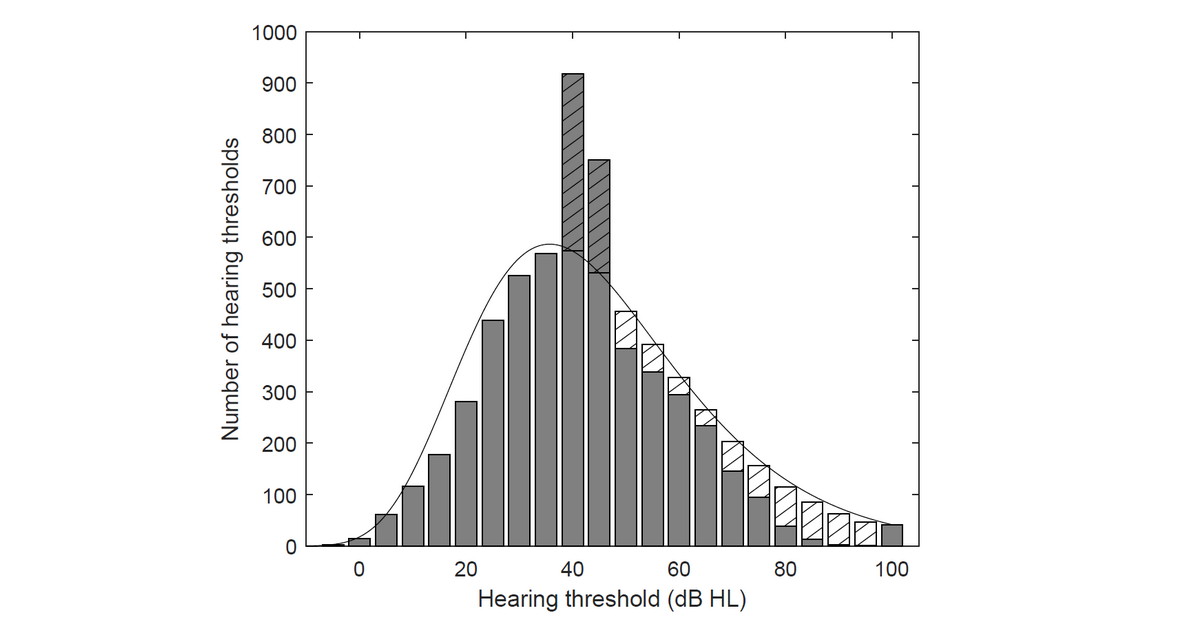
Hearing thresholds for subjects ≥60 years old at frequencies ≥4 kHz. The striped areas indicate the bias related to the masking noise that was enabled for stimuli >40 dB hearing level (HL). A lognormal distribution was assumed.

Hearing thresholds for older groups at a frequency ≥4 kHz are lower than those reported in the literature (Figure 3). A histogram for these groups reveals an increased number of hearing thresholds at intensities of 40 dB HL and 45 dB HL (Figure 8). This bias could be related to the masking noise. Contralateral masking noise was switched on when the signal level exceeded 40 dB HL. Thus, some of the subjects could react incorrectly to the masking noise instead of the test signal. The higher the mean threshold in a group at a given frequency, the more subjects might react incorrectly to the masking noise, thus leading to an understatement of the threshold. The lapses are rare for a low mean, and the bias is not detectable until the mean threshold reaches 40 dB. Therefore, the bias is observable in a limited range at 4 kHz (older groups only) and is unnoticeable at lower frequencies. Consequently, its impact on the average hearing threshold at 0.5-4 kHz is negligible, and the prevalence of hearing loss largely coincides with data reported in the literature. Nonetheless, in the future, changes should be introduced to the app that will prevent improper reaction to the masking noise.

#### Ambient Noise

Ambient noise was recorded using a built-in microphone with a working range of 72-102 dB SPL, as guaranteed by the Android specification [29]. Since the measurements were carried out outside this range, they should be treated as approximate. Nevertheless, the results obtained are comparable with data reported in the literature. Elevation of the hearing threshold as a result of an increase in ambient noise from 35 dB LAeq to 60dB LAeq was estimated at 8.0 dB (Figure 6), which agrees with the elevation of about 7.5 dB presented by Na et al [53].

A relatively low elevation of the hearing threshold related to a considerable increase in ambient noise may be associated with the measurement method applied in this study and in the study by Na et al [53]. Instead of being ready for the test signal during the whole test, the subject decides for herself or himself about the duration of the test signal presentation and can extend it during an event of louder noise. Subjects who spent sufficient time performing the test were able to assess the quietest signals during a temporary decrease in ambient noise.

The effect of ambient noise on hearing test results was determined assuming that the increase in hearing threshold caused by ambient noise is conditioned by the masking of the test signal by the noise. However, for significant hearing losses, ambient noise values above the normative level need not mask the loud test signal. Therefore, it must be assumed that the effect is present only for soft test signals and the estimated level for the effect of 3.78 dB (SD 0.75 dB) found in the present study should actually be treated as its upper limit. For these reasons, the results broadly correspond to pure-tone audiometry (Figure 3) despite ambient noise often exceeding the normative value for the audiometry.

#### Calibration

Mobile devices were calibrated using the biological method (ie, in relation to subjects with normal hearing). This method is less accurate than laboratory calibration. However, it enables semi-automatic calibration, which is crucial for as many as 1336 models. Because the tests were conducted on diverse device models in each country, the error associated with calibration has limited impact on differences between country-specific hearing loss prevalences.

### Conclusions

This paper presents global country-specific prevalences of hearing loss based on self-tests carried out by Android users. Unsupervised self-tests require additional quality control based on the test duration and connection status of the headphones. Moreover, ambient noise and calibration method introduce additional bias. Despite this, hearing thresholds measured by means of mobile devices were congruent with data reported in the literature, whereas uniform recruitment criteria facilitate the comparison between countries. Hearing tests on mobile devices may be a valid tool in epidemiological studies carried out on a large scale.

## Appendix

Multimedia Appendix 1 Median hearing threshold by age groups and by frequencies.

Multimedia Appendix 2 The prevalence of the hearing loss by countries.

Multimedia Appendix 3 Prevalence of the hearing loss in comparison with other studies.

## Abbreviations

HL: hearing level.

## References

[1] Mathers C, Smith A, Concha M (2003). Global burden of hearing loss in the year 2000. Global burden of Disease 2000.

[2] Stevens G, Flaxman S, Brunskill E, Mascarenhas M, Mathers CD, Finucane M, Global Burden of Disease Hearing Loss Expert Group (2013). Global and regional hearing impairment prevalence: an analysis of 42 studies in 29 countries. Eur J Public Health.

[3] GBD 2015 DiseaseInjury IncidencePrevalence Collaborators (2016). Global, regional, and national incidence, prevalence, and years lived with disability for 310 diseases and injuries, 1990-2015: a systematic analysis for the Global Burden of Disease Study 2015. Lancet.

[4] Wilson BS, Tucci DL, Merson MH, O'Donoghue GM (2017). Global hearing health care: new findings and perspectives. The Lancet.

[5] (2018). Addressing the rising prevalence of hearing loss.

[6] Arlinger S (2003). Negative consequences of uncorrected hearing loss--a review. Int J Audiol.

[7] Heine C, Browning CJ (2002). Communication and psychosocial consequences of sensory loss in older adults: overview and rehabilitation directions. Disabil Rehabil.

[8] Pearson JD, Morrell CH, Gordon-Salant S, Brant LJ, Metter EJ, Klein LL, Fozard JL (1995). Gender differences in a longitudinal study of age-associated hearing loss. J Acoust Soc Am.

[9] Mościcki EK, Elkins EF, Baum HM, McNamara PM (1985). Hearing loss in the elderly: an epidemiologic study of the Framingham Heart Study Cohort. Ear Hear.

[10] Cruickshanks KJ, Wiley TL, Tweed TS, Klein BE, Klein R, Mares-Perlman JA, Nondahl DM (1998). Prevalence of hearing loss in older adults in Beaver Dam, Wisconsin. The Epidemiology of Hearing Loss Study. Am J Epidemiol.

[11] Nash SD, Cruickshanks KJ, Klein R, Klein BEK, Nieto FJ, Huang GH, Pankow JS, Tweed TS (2011). The prevalence of hearing impairment and associated risk factors: the Beaver Dam Offspring Study. Arch Otolaryngol Head Neck Surg.

[12] Olusanya BO, Neumann KJ, Saunders JE (2014). The global burden of disabling hearing impairment: a call to action. Bull World Health Organ.

[13] Roth TN, Hanebuth D, Probst R (2011). Prevalence of age-related hearing loss in Europe: a review. Eur Arch Otorhinolaryngol.

[14] Foulad A, Bui P, Djalilian H (2013). Automated audiometry using apple iOS-based application technology. Otolaryngol Head Neck Surg.

[15] Kam ACS, Sung JKK, Lee T, Wong TKC, van Hasselt A (2012). Clinical evaluation of a computerized self-administered hearing test. Int J Audiol.

[16] Yousuf Hussein S, Wet Swanepoel D, Biagio de Jager L, Myburgh HC, Eikelboom RH, Hugo J (2016). Smartphone hearing screening in mHealth assisted community-based primary care. J Telemed Telecare.

[17] Renda L, Selçuk ÖT, Eyigör H, Osma Ü, Yılmaz MD (2016). Smartphone Based Audiometric Test for Confirming the Level of Hearing; Is It Useable in Underserved Areas?. J Int Adv Otol.

[18] Swanepoel DW, Myburgh HC, Howe DM, Mahomed F, Eikelboom RH (2014). Smartphone hearing screening with integrated quality control and data management. Int J Audiol.

[19] Sandström J, Swanepoel DW, Carel Myburgh H, Laurent C (2016). Smartphone threshold audiometry in underserved primary health-care contexts. Int J Audiol.

[20] Chu Y, Cheng Y, Lai Y, Tsao Y, Tu T, Young ST, Chen T, Chung Y, Lai F, Liao W (2019). A Mobile Phone-Based Approach for Hearing Screening of School-Age Children: Cross-Sectional Validation Study. JMIR Mhealth Uhealth.

[21] Ratanjee-Vanmali H, Swanepoel DW, Laplante-Lévesque A (2020). Patient Uptake, Experience, and Satisfaction Using Web-Based and Face-to-Face Hearing Health Services: Process Evaluation Study. J Med Internet Res.

[22] Sandström J, Swanepoel D, Laurent C, Umefjord G, Lundberg T (2020). Accuracy and Reliability of Smartphone Self-Test Audiometry in Community Clinics in Low Income Settings: A Comparative Study. Ann Otol Rhinol Laryngol.

[23] Bright T, Pallawela D (2016). Validated Smartphone-Based Apps for Ear and Hearing Assessments: A Review. JMIR Rehabil Assist Technol.

[24] Masalski M, Grysiński T, Kręcicki T (2018). Hearing Tests Based on Biologically Calibrated Mobile Devices: Comparison With Pure-Tone Audiometry. JMIR Mhealth Uhealth.

[25] Masalski M, Kipiński L, Grysiński T, Kręcicki T (2016). Hearing Tests on Mobile Devices: Evaluation of the Reference Sound Level by Means of Biological Calibration. J Med Internet Res.

[26] Masalski Marcin Hearing Test App.

[27] Masalski M, Grysiński T, Kręcicki T (2014). Biological calibration for web-based hearing tests: evaluation of the methods. J Med Internet Res.

[28] Masalski M, Kręcicki T (2013). Self-test web-based pure-tone audiometry: validity evaluation and measurement error analysis. J Med Internet Res.

[29] Audio: Implementation: Configuring Preprocessing Effects. Android Interfaces and Architecture.

[30] geoPlugin.

[31] Johansson MSK, Arlinger SD (2002). Hearing threshold levels for an otologically unscreened, non-occupationally noise-exposed population in Sweden. Int J Audiol.

[32] (2011). Recommended procedure: Pure-tone air-conduction and bone-conduction threshold audiometry with and without masking.

[33] Garg S, Kohli C, Mangla V, Chadha S, Singh MM, Dahiya N (2018). An Epidemiological Study on Burden of Hearing Loss and Its Associated Factors in Delhi, India. Ann Otol Rhinol Laryngol.

[34] (2017). Infant Mortality Rate. The World Factbook.

[35] (2000). ISO 7029:2000 Acoustics — Statistical distribution of hearing thresholds as a function of age.

[36] Kim S, Lim EJ, Kim HS, Park JH, Jarng SS, Lee SH (2010). Sex Differences in a Cross Sectional Study of Age-related Hearing Loss in Korean. Clin Exp Otorhinolaryngol.

[37] Engdahl B, Tambs K, Borchgrevink HM, Hoffman HJ (2005). Screened and unscreened hearing threshold levels for the adult population: results from the Nord-Trøndelag Hearing Loss Study. Int J Audiol.

[38] Strauss S, Swanepoel DW, Becker P, Eloff Z, Hall JW (2014). Noise and age-related hearing loss: a study of 40 123 gold miners in South Africa. Int J Audiol.

[39] Homans NC, Metselaar RM, Dingemanse JG, van der Schroeff MP, Brocaar MP, Wieringa MH, Baatenburg de Jong RJ, Hofman A, Goedegebure A (2017). Prevalence of age-related hearing loss, including sex differences, in older adults in a large cohort study. Laryngoscope.

[40] Lohi V, Ohtonen P, Aikio P, Sorri M, Mäki-Torkko E, Hannula S (2017). Hearing impairment is common among Saami adults in Northern Finland. Int J Circumpolar Health.

[41] Jun HJ, Hwang SY, Lee SH, Lee JE, Song J, Chae S (2015). The prevalence of hearing loss in South Korea: data from a population-based study. Laryngoscope.

[42] Mattos LC, Veras RP (2007). The prevalence of hearing loss in an elderly population in Rio de Janeiro: a cross-sectional study. Brazilian Journal of Otorhinolaryngology.

[43] Agrawal Y, Platz EA, Niparko JK (2008). Prevalence of hearing loss and differences by demographic characteristics among US adults: data from the National Health and Nutrition Examination Survey, 1999-2004. Arch Intern Med.

[44] Helzner EP, Cauley JA, Pratt SR, Wisniewski SR, Zmuda JM, Talbott EO, de Rekeneire N, Harris TB, Rubin SM, Simonsick EM, Tylavsky FA, Newman AB (2005). Race and sex differences in age-related hearing loss: the Health, Aging and Body Composition Study. J Am Geriatr Soc.

[45] Louw C, Swanepoel DW, Eikelboom RH, Hugo J (2018). Prevalence of hearing loss at primary health care clinics in South Africa. Afr Health Sci.

[46] Lin FR, Thorpe R, Gordon-Salant S, Ferrucci L (2011). Hearing loss prevalence and risk factors among older adults in the United States. J Gerontol A Biol Sci Med Sci.

[47] Sindhusake D, Mitchell P, Smith W, Golding M, Newall P, Hartley D, Rubin G (2001). Validation of self-reported hearing loss. The Blue Mountains Hearing Study. Int J Epidemiol.

[48] Lin C, Yang Y, Guo YL, Wu C, Chang C, Wu J (2007). Prevalence of hearing impairment in an adult population in Southern Taiwan. Int J Audiol.

[49] Lin FR, Niparko JK, Ferrucci L (2011). Hearing loss prevalence in the United States. Arch Intern Med.

[50] Fisher DE, Li C, Hoffman HJ, Chiu MS, Themann CL, Petersen H, Jonsson PV, Jonsson H, Jonasson F, Sverrisdottir JE, Launer LJ, Eiriksdottir G, Gudnason V, Cotch MF (2015). Sex-specific predictors of hearing-aid use in older persons: The age, gene/environment susceptibility - Reykjavik study. Int J Audiol.

[51] Brennan-Jones CG, Taljaard DS, Brennan-Jones SEF, Bennett RJ, Swanepoel DW, Eikelboom RH (2016). Self-reported hearing loss and manual audiometry: A rural versus urban comparison. Aust J Rural Health.

[52] Zhan W, Cruickshanks KJ, Klein BEK, Klein R, Huang G, Pankow JS, Gangnon RE, Tweed TS (2010). Generational differences in the prevalence of hearing impairment in older adults. Am J Epidemiol.

[53] Na Y, Joo HS, Yang H, Kang S, Hong SH, Woo J (2014). Smartphone-based hearing screening in noisy environments. Sensors (Basel).

