# Supplementary figures and images for: Worldwide Prevalence of Hearing Loss Among Smartphone Users: Cross-Sectional Study Using a Mobile-Based App
Source: J Med Internet Res. 2020 Jul 23;22(7):e17238. doi: 10.2196/17238 (PMC7413293; doi:10.2196/17238)

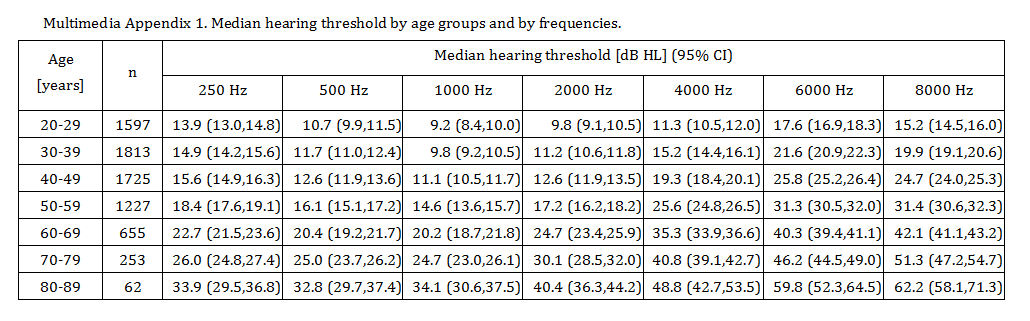

Supplement: Multimedia Appendix 1 [file jmir_v22i7e17238_app1.png]

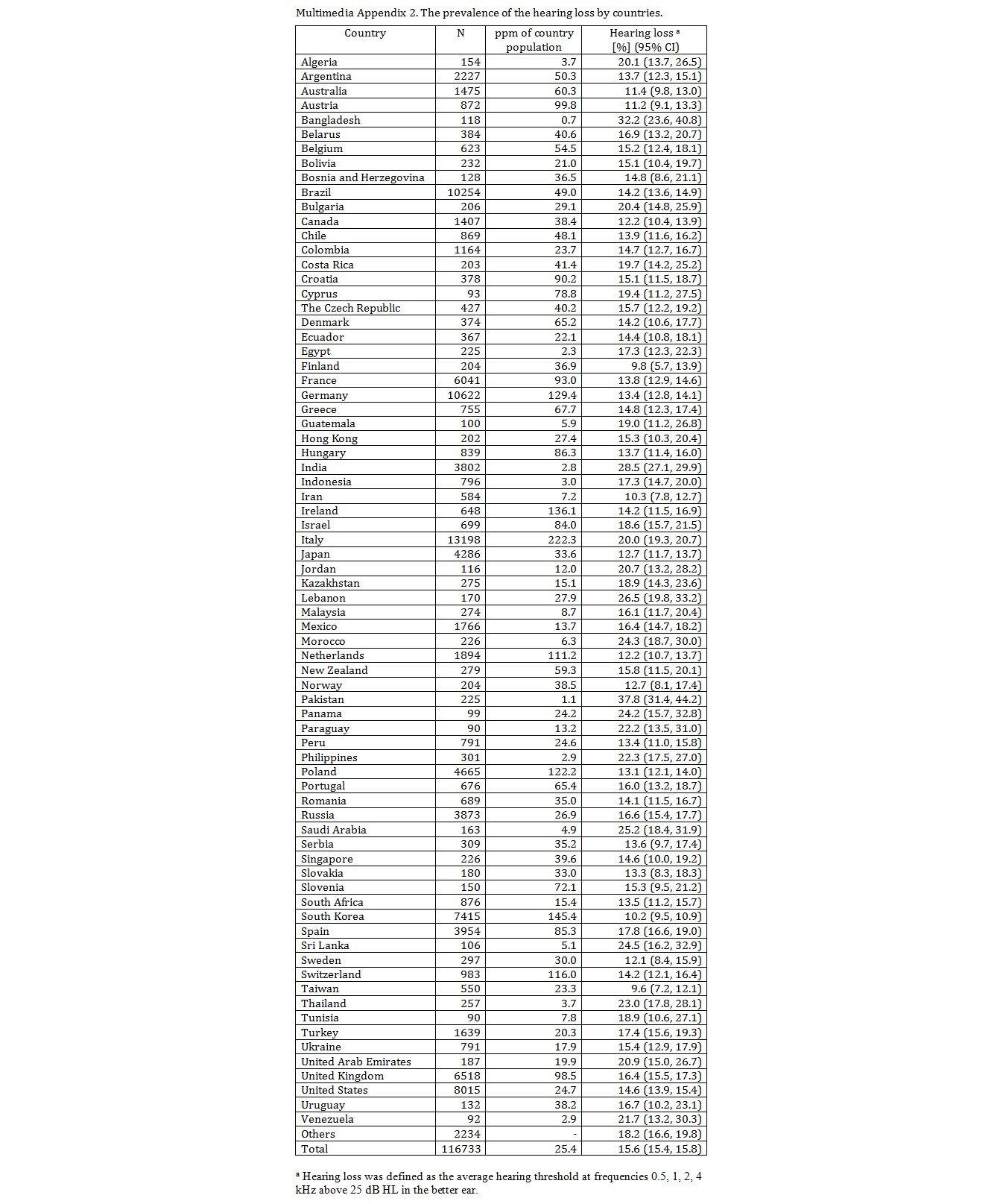

Supplement: Multimedia Appendix 2 [file jmir_v22i7e17238_app2.png]

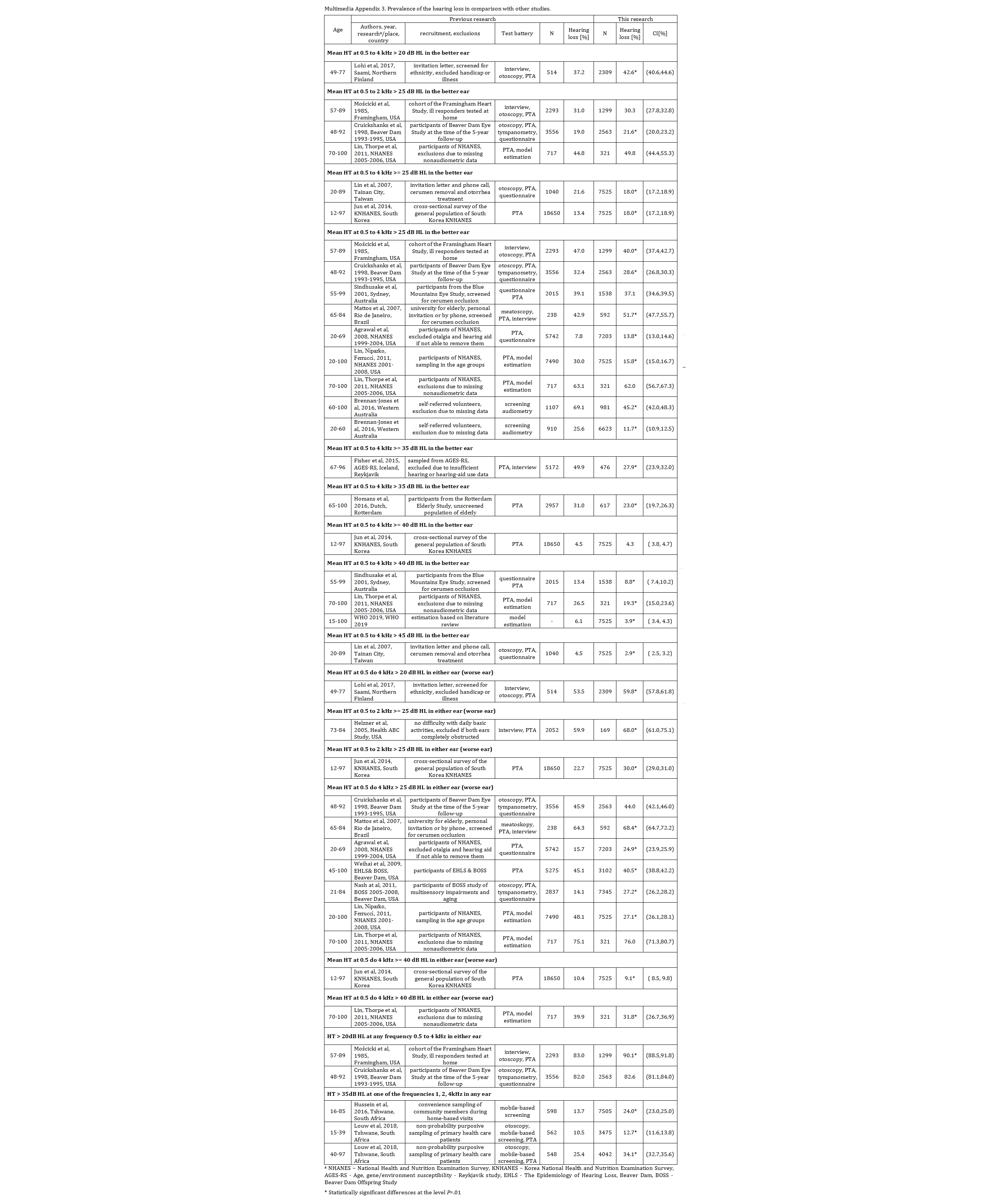

Supplement: Multimedia Appendix 3 [file jmir_v22i7e17238_app3.png]
